# Tartrate-resistant acid phosphate as biomarker of bone turnover over the lifespan and different physiologic stages in sheep

**DOI:** 10.1186/s12917-017-1170-9

**Published:** 2017-08-15

**Authors:** José A. Camassa, Camila C. Diogo, João P. A. Bordelo, Marília de A. Bonelli, Carlos A. Viegas, Jorge T. Azevedo, Nuno Dourado, Isabel R. Dias

**Affiliations:** 1 Department of Veterinary Sciences, Agricultural and Veterinary Sciences School (ECAV), University of Trás-os-Montes and Alto Douro (UTAD), Quinta de Prados, P.O. Box 1013, 5001-801 Vila Real, Portugal; 2 Federal Rural University of Pernambuco (UFRPE), R. Dom Manoel de Medeiros, s/n, Dois Irmãos, Recife, PE 52171-900 Brazil; 3 CITAB – Centre for the Research and Technology of Agro-Environmental and Biological Sciences, UTAD, Vila Real, Portugal; 4 Department of Animal Sciences, ECAV, UTAD, Vila Real, Portugal; 5 CECAV – Centre for Animal Sciences and Veterinary Studies, UTAD, Vila Real, Portugal; 6 CMEMS-UMinho, Department of Mechanical Engineering, University of Minho, Campus de Azurém, 4804-533 Guimarães, Portugal

**Keywords:** ALP, Bone tissue, Serum minerals, Sheep, Tartrate-resistant acid phosphatase

## Abstract

**Background:**

Currently, the best resources for assessment of bone tissue using imaging techniques are expensive and available in few medical facilities, thus serum or urinary bone turnover biomarkers could be useful as early indicators of prognosis. However, there is a wide range of variability in bone turnover markers due to several factors, such as different ages and metabolic stages, thus it is important to have as much data published on the subject as possible. The aim of this study was therefore to generate a reference range for alkaline phosphatase (ALP) and tartrate-resistant acid phosphatase (TRAP) and validate the already published data.

**Results:**

Serum alkaline phosphatase decreased with age, with statistical difference between the 1 month old and the other groups and between the over 8 years and the 6 months old groups. There was also a statistical difference in the ALP levels between the 3 to 5 years old gestation and lactation groups. For serum tartrate-resistant acid phosphatase, there was a statistical difference mainly between the 1 month old and the 6 months old, 6–8 years old, and above 8 years old groups.

**Conclusions:**

The results obtained could represent a useful tool for future studies using sheep as an animal model for orthopedic research. The different groups presented a wide variation of serum ALP and TRAP levels, however, these variations are entirely explained by known physiology. Therefore, this detailed study confirms the prediction that unexplained changes in these bone turnover markers do not occur during the lifespan of sheep.

## Background

Currently, skeletal and bone tissue assessment is performed by diagnostic imaging such as radiography, computed tomography, magnetic resonance and densitometry techniques, or invasive methods, such as bone biopsy, although these methods are still limited in many situations. Therefore, determination of the serum levels or urinary bone turnover biomarkers (BTMs) could be a good noninvasive analytical method for evaluation of bone metabolism, as it allows predicting bone cell activity in a quick and real time manner [1].

At present, some studies have been performed on BTMs in animal models for orthopedic research not only to improve the knowledge in animal and veterinary sciences, but also in human orthopedic research of metabolic bone diseases, such as post-menopausal osteoporosis. BTMs could be useful in diagnosing early-stage bone diseases, and helping to monitor evolution and efficiency of treatment.

BTMs are generally divided in two groups: the formation and the bone resorption markers, although there is a third group that is still poorly studied, the osteoclast regulatory protein [2]. During the metabolic process of bone formation by osteoblasts, formation markers are represented by serum alkaline phosphatase (ALP) and its bone-specific isoform (BALP), serum osteocalcin, and two other molecules that are released during synthesis of the type I collagen molecule **–** serum procollagen type I carboxy- and amino-terminal propeptides [3].

The ALP is a glycoprotein that is connected to the surface of cells. In humans ALP are expressed in four gene loci code: nonspecific, intestinal, placental and germ cells [4]. Nonspecific gene is synthesized in a variety of tissues (bone, kidney, liver and early placenta) [4]. BALP has been used due to its high sensitivity as a bone formation marker [4]. It is produced by osteoblasts [5] and is involved in the calcification of bone matrix [6].

In the bone resorption process, there is a breakdown of type I collagen, so resorption markers are represented by serum C-terminal telopeptide of type I collagen, urinary collagen type I cross-linked C- and N-telopeptide, urinary hydroxyproline, total and free urinary pyridinoline and deoxypyridinoline, as well as by the serum tartrate-resistant acid phosphatase (TRAP), which is produced by active osteoclasts [7].

TRAP is a glycoprotein produced by mature osteoclasts, activated dendritic cells, and macrophages, therefore TRAP is an indicator of osteoclast and macrophage activity [8, 9]. There are two known isoforms (TRAP5a,b). TRAP5b is a specific biomarker of osteoclastic resorption activity, while TRAP5a is a non-osteoclastic form [10]. In the future, the latter may be useful in clinical evaluation since it is expressed in bone pathologies, such as fracture healing in different mammals or osteoporosis in women [9, 11, 12].

The use of sheep as animal models in orthopedic research, also in studies focusing on BTMs, is based on different aspects such as easy handling [13, 14] and possibility of blood sample collection several times per day [15] due to a high blood volume when compared to other laboratory animal species. Nevertheless, bone markers in small ruminants are influenced by circadian and seasonal variation [16], among other possible factors causing serum and urinary variability such as diet, exercise, skeletal growth, as well as intra-individual variability [4]. As such, BTMs undergo variations throughout a lifetime, namely with age and different metabolic states. It is therefore important to assess BTMs variation during these phases and establish a reference range for BTMs in this species, to which the greatest limitation is the inter-individual variability [17].

Therefore, the aim of this study was to generate a reference range for some of the main BTMs – ALP and TRAP, throughout the lifespan and different physiological states in sheep and to evaluate possible correlations of these parameters with serum minerals.

## Methods

### Animals

Ninety ewes (Churra-da-Terra-Quente sheep) from the same flock, located in Carrazeda de Ansiães, a municipality in the district of Bragança in northern Portugal were used. Average minimum temperature was 1.9 °C and average maximum temperature 10.2 °C in December at Bragança. Sheep were kept in a natural pasture during the day and housed overnight. The barn is easily approachable and spacious, dry, well-drained, well-ventilated and bedding composed by hay and straw. These animals were chosen among a flock according to their age or physiologic state and divided into 9 groups of 10 animals each. The groups were as follows: 1 month old (mean weight 9.3 kg), 6 months old (mean weight 22.2 kg), 1 year old (mean weight 40.7 kg), 2 years old (mean weight 50.2 kg), 3 to 5 years old dry (mean weight 52.4 kg), 3 to 5 years old with 2 or 3 months of pregnancy (mean weight 55.3 kg), 3 to 5 years old with 2 or 3 months of lactation (mean weight 51.5 kg), 6 to 8 years old (mean weight 52.1 kg), and the last group with animals over 8 years old (mean weight 48.1 kg). The diet was composed by grass hay, supplemented with 0.250 kg of concentrate feed per animal per day and water provided ad libitum. Dry matter and chemical composition of grass hay is made up of dry matter per kg feed (88.5 g), ash per kg dry matter (5.9 g), neutral detergent fiber per kg (73.3 g) dry matter and crude protein per kg dry matter (6.1 g). Dry matter and chemical composition of feed concentrate is made up of dry matter per kg feed (90.4 g), ash per kg dry matter (8.5 g), neutral detergent fiber per kg (31.6 g) dry matter and crude protein per kg dry matter (20.7 g).

All animal handling practices followed Directive 2010/63/EU of the European Parliament and of the Council on the protection of animals used for scientific purposes.

### Blood sampling

Blood was drawn in December during the European winter. Blood samples were drawn from the jugular vein and placed into serological tubes (S-Monovette®, SARSTEDT, Nümbrecht, Germany). Samplings were performed between 9:00 a.m. and 10:00 a.m. and the blood carried in a thermal box to laboratory facilities immediately. Blood was centrifuged (3000 rpm for 10 min) and the serum stored in Eppendorf tubes at −20 °C until analyses.

### Serum biochemical analysis

Following the manufacturer’s instructions, the assays were always performed in duplicate to achieve higher accuracy.

Determination of ALP (Alkaline Phosphatase, Beckman Coulter, Ref. OSR6004, CA, USA) and TRAP (ACP, Ref. 17,617; Sentinel Diagnostics, Milan, Italy) were performed via an enzymatic method and molecular absorption spectrophotometry using commercially available kits. Calcium (Ca) (Calcium, Beckman Coulter, Ref. OSR60117, CA, USA), phosphorus (P) (Phosphorus, Beckman Coulter, Ref. OSR6122, CA, USA) and magnesium (Mg) (Magnesium, Beckman Coulter, Ref. OSR6189, CA, USA) were also determined using commercially available kits, via chemical method and molecular absorption spectrophotometry.

### Statistical analysis

Statistical normality was checked using the Shapiro-Wilk W-Test for all groups. Serum BTMs and mineral values are presented as median ± interquartile range (IQR), minimum (Min) and maximum (Max). Spearman’s correlation was obtained between the serum biochemical markers. The Kruskal - Wallis Test was used for testing the non-parametric statistical hypothesis and the Kruskal – Wallis pairwise method for multiple comparisons. Statistical analysis was performed using SPSS software (version 23.0, SPSS, Inc., IBM Company, NY, USA). The level of significance was set at *P* < 0.05.

## Results

All parameters in this study revealed a non-normal distribution for age analysis. The median and interquartile range for each marker in different ages are shown in Table 1.

**Table 1:** Values of serum biochemical markers and serum minerals by ages

		Median ± IQR	Range
			(Min–Max)
ALP (U/L)	1 month	849.5 ± 270.7	520–1354
	6 months	269.5 ± 136.2	89–454
	1 year	220.0 ± 135.0	138–583
	2 years	200.5 ± 90.25	116–295
	3–5 years gestation	104.5 ± 116.0	66–350
	3–5 years lactation	330.5 ± 159.2	105–390
	3–5 years dry	181.0 ± 133.5	108–363
	6–8 years	197.0 ± 127.0	63–444
	>8 years	119.0 ± 177.0	35–277
TRAP (U/L)	1 month	3.15 ± 0.45	2.9–3.7
	6 months	2.65 ± 0.10	2.5–2.9
	1 year	3.10 ± 0.50	2.4–3.6
	2 years	2.95 ± 0.60	2.2–3.2
	3–5 years gestation	2.80 ± 0.25	2.6–3.0
	3–5 years lactation	2.55 ± 0.37	2.3–2.8
	3–5 years dry	2.80 ± 0.25	2.0–3.3
	6–8 years	2.50 ± 0.07	2.4–2.7
	>8 years	2.65 ± 0.47	1.6–3.3
Calcium (mmol/L)	1 month	2.83 ± 0.12	2.57–2.90
	6 months	2.59 ± 0.23	2.35–2.75
	1 year	2.63 ± 0.10	2.55–2.83
	2 years	2.53 ± 0.20	2.33–2.68
	3–5 years gestation	2.49 ± 0.16	2.18–2.68
	3–5 years lactation	2.49 ± 0.18	2.38–2.65
	3–5 years dry	2.54 ± 0.13	2.35–2.65
	6–8 years	2.61 ± 0.10	2.50–2.68
	>8 years	2.33 ± 0.21	1.85–2.48
Magnesium (mmol/L)	1 month	0.91 ± 0.07	0.78–1.03
	6 months	0.96 ± 0.04	0.96–1.04
	1 year	1.02 ± 0.14	0.92–1.29
	2 years	1.04 ± 0.09	0.95–1.12
	3–5 years gestation	1.08 ± 0.17	0.92–1.29
	3–5 years lactation	1.03 ± 0.03	1.02–1.08
	3–5 years dry	0.97 ± 0.07	0.93–1.13
	6–8 years	1.05 ± 0.13	0.89–1.32
	>8 years	1.12 ± 0.26	0.88–1.56
Phosphorous (mmol/L)	1 month	3.21 ± 0.27	3.10–3.52
	6 months	2.21 ± 0.53	1.06–2.87
	1 year	1.87 ± 0.46	1.58–2.29
	2 years	1.59 ± 0.25	1.23–1.81
	3–5 years gestation	1.42 ± 0.63	1.13–1.98
	3–5 years lactation	1.45 ± 0.30	1.16–1.81
	3–5 years dry	1.36 ± 0.24	0.90–1.90
	6–8 years	1.56 ± 0.36	1.10–2.06
	>8 years	1.64 ± 0.05	1.06–2.42

ALP: alkaline phosphatase; TRAP: tartrate-resistant acid phosphatase; IQR: interquartile range; Min: minimum; Max: maximum

**Fig. 1:**
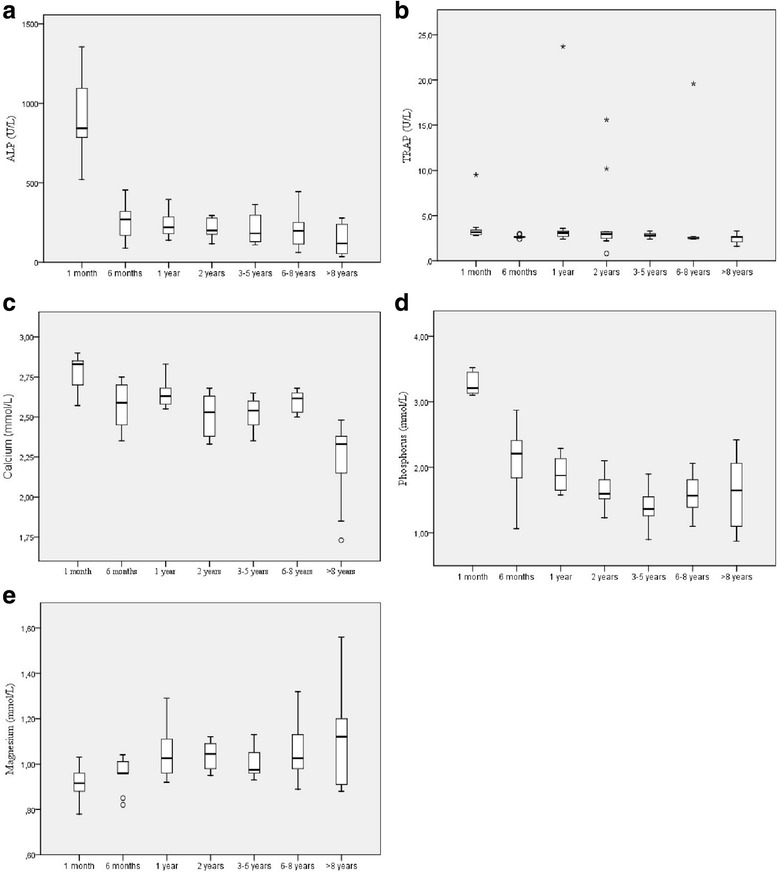
Box plot presentations of serum concentrations of biomarkers of bone metabolism by age. **a** Serum ALP activity presented significant difference between 1 month vs. 6 months (*P* < 0.01), between 1 month vs. 1 year, 2 years, 3–5 years, 6–8 years and >8 years (*P* < 0.001), between 6 months vs. >8 years (*P* < 0.05). **b** Serum TRAP activity presented significant difference between 1 month vs. 6 months, >8 years (*P* < 0.01), between 1 month vs. 6–8 years (*P* < 0.001), between 1 year vs. 6 months, >8 years (*P* < 0.05), between 1 year vs. 6–8 years (*P* < 0.01), between 2 years vs. 6–8 years (*P* < 0.05). **c** Serum calcium activity presented significant difference between 1 month vs. 6 months, 6–8 years (*P* < 0.05), between 1 month vs. 2 years and 3–5 years (*P* < 0.01), between 1 month vs. >8 years (*P* < 0.001), between >8 years vs. 2 years and 3–5 years (*P* < 0.05), between >8 years vs. 6 month and 6–8 years (*P* < 0.01) and between >8 years vs. 1 year (*P* < 0.001). **d** Serum ALP activity presented significant difference between 1 month vs. 6 months (*P* < 0.05), between 1 month vs. 1 year (*P* < 0.01), between 1 month vs. 2 years, 3–5 years, 6–8 years and >8 years (*P* < 0.001), between 6 months vs. 2 years, 3–5 years, 6–8 years and >8 years (*P* < 0.05) and between 1 year vs. 3–5 year (*P* < 0.05). **e** Serum Magnesium activity presented significant difference between 1 month vs. 3–5 years (*P* < 0.05) and between 1 month vs. 1 year, 2 years, 6–8 years and >8 years (*P* < 0.01). Outliers are identified with small circle for out values and star for extreme values

Figure 1a shows a significant difference between the 1 month old group and all the other groups, and a significant difference between animals of 6 months of age and over 8 years for ALP (Fig. 1). For TRAP, the 1 month old and 1 year old groups were the ones with a significant statistical difference from the other groups (Fig. 1b).

Calcium and phosphorus both suffer a slight decrease throughout the animal’s life, with a statistical difference seen in the 1 month old and over 8 years old groups for Ca (Fig 1c), and 6 months old and 1 month old groups for P (Fig. 1d), with the latter showing a significant difference when compared to all other groups except the 6 months old group. Magnesium had a slight increase with age (Fig. 1e), with the most significant difference observed between the 1 month old group and the other groups. The degrees of correlation are shown in Table 2. The results expressed a fair correlation between markers, with the highest correlation observed between ALP and P (*r* < 0.60; *P* < 0.01).

**Table 2:** Correlation between serum biochemical markers and serum minerals

	ALP	TRAP	Ca	P	Mg
ALP	-	*r* = 0.44^b^	*r* = 0.514^b^	*r* = 0.581^b^	*r* = 0.229
TRAP	-	-	*r* = 0.379^b^	*r* = 0.261^a^	*r* = −0.066
Ca	-	-	-	*r* = 0.492^b^	*r* = −0.322^b^
P	-	-	-	-	*r* = −0.289^a^
Mg	-	-	-	-	-

^a^ *correlation* coefficient is *significant* at the *0.05* level

^b^ *correlation* coefficient is *significant* at the *0.01* level

**Fig. 2:**
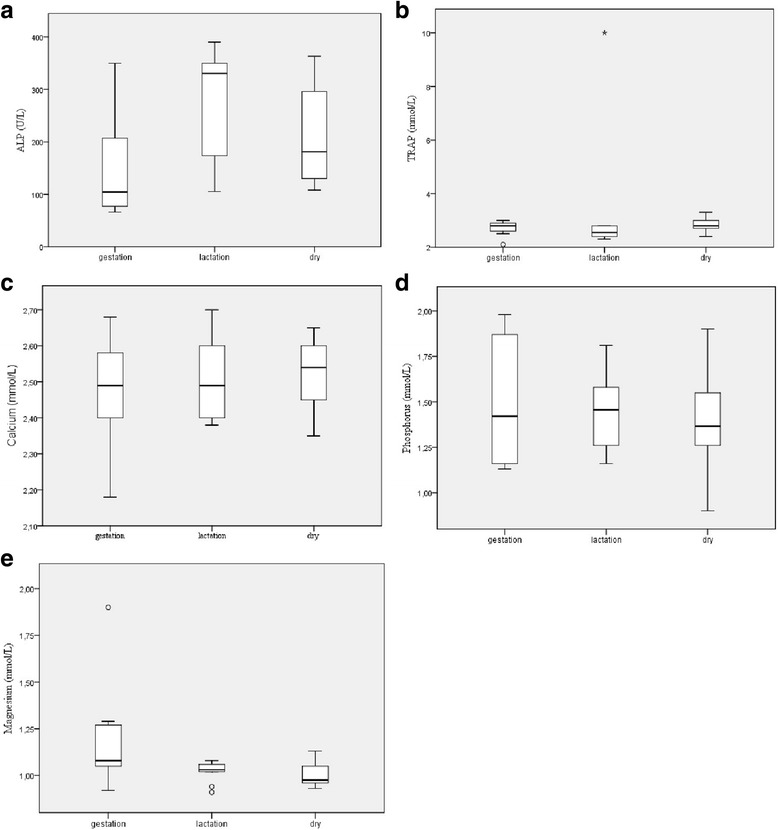
Box plot presentations of serum concentrations of biomarkers of bone metabolism by physiologic stages. **a** Serum ALP activity presented significant difference between gestation vs. lactation (*P* < 0.05). **b** Serum TRAP activity, there was no significant difference between the groups. **c** Serum Calcium activity, there was no significant difference between the groups. **d** Serum Phosphorus activity, there was no significant difference between the groups. **e** Serum Magnesium activity, there was no significant difference between the groups. Outliers are identified with small circle for out values and star for extreme values

In the analyses of the groups with animals between 3 and 5 years of age in different physiologic stages, ALP had a significant difference between the gestation and lactation groups (Fig. 2a) and TRAP (Fig. 2b) had a significant difference between the dry and lactation groups. Normality analyses of the groups with 3 to 5 years of age had a normal distribution for Ca and P (Fig. 2c and d), ALP, TRAP and Mg (Fig. 2e) had a non-normal distribution. Correlation between the three groups did not reveal a statistically significant difference.

## Discussion

The aim of this study was to assess the behavior of serum ALP, TRAP, and minerals in different ages and physiologic stages of the ewes life, therefore providing a dataset and information to assist in research studies focusing on BTMs in sheep.

In this study, the median ALP value was within the normal range reported for this species (68–387 U/L) [18] and the variations over the lifespan in sheep can explained by physiological changes. The interval range was wide, however, possibly due to such factors as seasonal influence, with decreased formation marker (BALP) during Autumn [16], or circadian influence, in which there is a variation of bone markers throughout the day. Because of the latter, blood samples sampling must be drawn at a standardized time [15]. Age was another influencing factor, with a higher level of ALP found in 1-month old animals, possibly due to synthesis in a variety of tissues, not only due to the bone isoenzyme [4], and high juvenile metabolism [19]. Lower levels of ALP were found in sheep over 8 years of age, possibly due to the influence of age in decreasing bone formation. In another study, Haversian remodelling in the caudal aspect of the femur, diaphysis of the radius and humerus was observed in sheep between 7 and 9 years of age [20], which justifies a link between advanced age and bone loss.

Previously reported values for P (1.62–3.36 mmol/L) and Mg (0.9–1.31 mmol/L) [18] were within the interval found in the present study, although Ca levels (2.88–3.2 mmol/L) [18] were higher in the aforementioned paper. This Ca value could be caused by a decrease in this mineral in pasture, as previously described [21]. In the referred works the lowest value of Ca was found in different samples from different grazing areas during Winter. Blood samples in the present study were drawn in the same season, however, pasture analyses were not performed which renders impossible to investigate a correlation.

In general, for serum minerals, there was a significant difference between the 1 month old group and the other groups, possibly due to the high bone modelling process that is occurring at this age. In fact, as the skeletal structure in these animals is still in growth, there is an increasing demand for Ca and P [22]. A statistical difference in serum minerals between 1-month-old and 6-months-old groups was not expected since both groups have growing animals. There was, however, a statistical difference in Ca and P between the two groups, possibly due to a change in feed, with the absence of milk and a diet based on grass and concentrate at 6 months old. The decrease in Ca in animals over 8 years of age when compared to the other groups may be associated with bone remodelling in older sheep, as has been reported [20], where the decline in Ca would thus be expected due to a decrease in bone mineralization.

During the analysis of the TRAP marker, the 1-month old animals had a higher range interval, probably related to an accelerated resorption during skeletal growth [9]. However, 6-months-old animals had a low level of this marker even though the sheep were undergoing skeletal growth, possibly because of a difference in diet, as previously mentioned. This marker showed an inversely proportional relationship with age, thus the animals with 6 to 8 years of age had lower values among all the groups. However, those over 8 years old had an increased TRAP, possibly due to osteoclastic activity [9] in geriatric animals. An explanation for this increase has not been investigated in this study in older sheep. Nevertheless, in older women, it is associated to osteoporosis [23]. Therefore, there may be an osteopenia in old animals related to an increase of this marker, but analyses of the bone, such as bone densitometry, would be necessary to prove that.

It should also be mentioned that the ideal TRAP markers to assess bone metabolism should determine the TRAP5b isoform by itself, since it has an osteoclastic origin, resulting from resorption activity, whereas the TRAP5a isoform is nonosteoclastic [24]. The occurrence of seven outliers (three in 6 months old, one in the 2 years and three in 8 years old group) in the determination of TRAP could be justified in this study by the marker being expressed in different tissues, such as muscles and heart, or pathological conditions. There are published studies in humans that correlated TRAP with leukemia and AIDS [25]. However, the present study used healthy animals and obtained normal values for the ovine species. In the present study, the values found are within the reported range of minimum value in adults and maximum values in juveniles (0,14–5,9 U/L) [11]. It is thus not possible to state which tissue was responsible for the occurrence of outliers and whether there was a pathological cause for these outliers. All TRAP variations can be explained by physiological or pathological changes as previously described.

This study presented a reasonable degree of correlation between P and Ca, previously described in sheep as being involved in bone mineralization [26]. In rats, the increase of these serum minerals were essential for mineralization of bone tissue developed in vitro [27]. The degree of correlation present between ALP and Ca is possibly caused by the role of Ca in enzymatic reactions involving ALP, a correlation which has been previously described [3, 12].

With regard to the three groups of 3- to 5-year old sheep in different physiologic stages, only the pregnant group showed values that were considerably lower than the dry and lactation groups for ALP analyses. A similar increase of ALP throughout pregnancy has been previously described [28]. However, Liesegang et al. (2006) [29] used the BALP isoform and obtained a decrease of the marker during gestation. Therefore, when the animals become pregnant, there is a significant reduction of ALP, which then increases between gestation and lactation, and is slightly decreased in dry sheep [28]. In this study, the same marker variation was observed among the three described physiologic states, as has been previously described. However, during the analysis of TRAP, the lactating group showed lower values when compared to the dry ones. This could be due to the abrupt drop in bone resorption during the 2 to 3 months of lactation, with values going back to basal values in dry animals. Another greatly important factor would be that the maximum peak of TRAP would occur closer to parturition, as described [28].

## Conclusions

This study provided information on the variation of two of the most used BTMs – ALP and TRAP, throughout the different stages of life and metabolic stages in sheep. These bone turnover markers variations can be entirely explained by known physiology, confirming that unexplained changes do not occur during the lifespan of sheep. This information may be a useful tool in clinical orthopedic research studies both in animal and veterinary sciences, as well as when using sheep as an animal model for translational studies for humans.

## Abbreviations

ALP: Alkaline phosphatase.
BALP: Bone-specific isoform alkaline phosphatase.
BTMs: Bone turnover biomarkers.
Ca: Calcium
IQR: Interquartile range
Kg: Kilogram
Max: Maximum
Mg: Magnesium
Min: Minimum
P: Phosphorus
rpm: Revolutions per minute
TRAP5a: Tartrate-resistant acid phosphatase isoform 5a
TRAP5b: Tartrate-resistant acid phosphatase isoform 5b
TRAP: Tartrate-resistant acid phosphatase
